# Lack of association between celiac disease and dental enamel hypoplasia in a case-control study from an Italian central region

**DOI:** 10.1186/1746-160X-3-25

**Published:** 2007-05-30

**Authors:** Maurizio Procaccini, Giuseppina Campisi, Pantaleo Bufo, Domenico Compilato, Claudia Massaccesi, Carlo Catassi, Lorenzo Lo Muzio

**Affiliations:** 1 Istituto di Scienze Odontostomatologiche, Università Politecnica delle Marche, Italy; 2 Dip. Scienze Stomatologiche, Università di Palermo, Italy; 3 Dip. Scienze Chirurgiche, Università di Foggia, Italy; 4 Istituto di Clinica Pediatrica, Università Politecnica delle Marche, Italy

## Abstract

**Background:**

A close correlation between celiac disease (CD) and oral lesions has been reported. The aim of this case-control study was to assess prevalence of enamel hypoplasia, recurrent aphthous stomatitis (RAS), dermatitis herpetiformis and atrophic glossitis in an Italian cohort of patients with CD.

**Methods:**

Fifty patients with CD and fifty healthy subjects (age range: 3–25 years), matched for age, gender and geographical area, were evaluated by a single trained examiner. Diagnosis of oral diseases was based on typical medical history and clinical features. Histopathological analysis was performed when needed. Adequate univariate statistical analysis was performed.

**Results:**

Enamel hypoplasia was observed in 26% cases vs 16% in controls (p > 0.2; OR = 1.8446; 95% CI = 0.6886: 4.9414). Frequency of RAS in the CD group was significantly higher (36% vs 12%; p = 0.0091; OR = 4.125; 95% CI = 1.4725: 11.552) in CD group than that in controls (36% *vs *12%). Four cases of atrophic glossitis and 1 of dermatitis herpetiformis were found in CD patients *vs *1 and none, respectively, among controls.

**Conclusion:**

The prevalence of enamel hypoplasia was not higher in the study population than in the control group. RAS was significantly more frequent in patients with CD.

## Background

Celiac disease (CD), also known as celiac sprue or gluten-sensitive entheropathy, can be defined as a chronic inflammatory intestinal disease characterised by nutrient malabsorption and improvement after the withdrawal of gluten (found in wheat, barley) from the diet. Prevalence of CD ranges from 1:85 to 1:300 have been reported for CD in Western countries [1-6]. In addition to the classical gastrointestinal presentation (diarrhoea, abdominal distension, vomiting, weight loss and pallor) CD can cause minimal intestinal damage and weak or absent systemic symptomatology (also known as "silent form"). In these patients the lack of symptoms can persist for a long time, while the biopsy of the bowel shows the typical atrophy of intestinal mucosa [7]. It is also well recognized the association of CD with several complications, as lymphomas, autoimmune and degenerative nervous system diseases [8-10].

The oral cavity, a part of gastrointestinal system [11], can also be affected by several abnormalities in patients with CD. As the mouth is very easy to examine, oral lesions can provide a valuable clinical clue for early diagnosis of CD [12]; in fact among the atypical aspects of CD (extra-intestinals), in the international literature has been reported some affections interesting the oral cavity, the most common are recurrent aphthous stomatitis (RAS) [13-15] and dental enamel defects [8,13,16-21], in addition have been described the association between CD and unspecific forms of atrophic glossitis [22], oral manifestations of dermatitis herpetiformis [23], Sjögren's syndrome [24,25] and oral lichen planus [26,27]. These disorders, in absence of a typical intestinal symptomatology, can represent useful clues for a timely diagnosis [7,22].

However, data from literature are often controversial, probably because of different geographical origin of patients studied and lack of adequate controls. Finally, no studies have been performed, in CD patients of a Central Region of Italy (Ancona, Marche, Italy)

The aim of this case-control study was to assess prevalence of dental hard and oral soft tissues changes generally considered celiac-related (e.g. RAS, enamel hypoplasia, dermatitis herpetiformis and atrophic glossitis) and to verify if cases are more likely to be affected by any of the oral diseases considered.

## Methods

Fifty CD patients, aged between 3 and 18 years old and living in the Region of Marche, were enrolled in the study. CD was diagnosed at Paediatric Department of the University Politecnica of Marche (Ancona, Italy), and the diagnosis of CD was based on serological tests (Ab-htTG IgA, Ab-htTG IgG, AGA IgA, AGA IgG, EMA IgA, EMA IgG), small-bowel biopsy during esophago-gastro-duodenoscopy (EGDS) and histological evidence of villous atrophy with crypt hyperplasia and increase in intraepithelial lymphocytes (normal, 10–30 per 100 epithelial cells), and the disappearance of the symptoms and normalization of serum anti-tTG and/or EMA after gluten-free-diet (GFD) [28,29]. The control group was recruited by simple randomization at a Primary and Secondary Public School of Ancona, during an healthy prevention programme for oral disease, matched one-to-one and without any significant differences with study group for geographical area, age and gender (p > 0.2 by *t*-Student and chi-square test, respectively). These young individuals neither reported any gastrointestinal diseases and not have a family history of CD.

Patients were examined for hard tissue changes (i.e. dental enamel defects) and soft tissue lesions (RAS, dermatitis herpetiformis and atrophic glossitis). Patients with CD and healthy individuals were examined by a single observer. Informed consent was obtained by parents who were also asked about previous episodes of RAS affecting child/children.

The enamel defects affecting deciduous and permanent teeth were graded 0 to IV according to Aine's classification [17] with a special attention to symmetric anomalies.

Soft tissues examination was carried out with conventional dental chairs, artificial light, flat mirrors, monouse probe and sterile gauzes.

With regard RAS, we registered both lesions clinically observed and ulcerative events referred by parents or reported by hospital clinical records. They were classified into minor, major and herpetic aphthous ulcers [30], according to dimension, form, localization and evolutionary tendency, and also rate of occurrence was registered. Atrophic glossitis was diagnosed on the basis of clinical features and oral mucosal lesions due to dermatitis herpetiformis were assessed by both clinical features and histological/immunofluorescence studies.

### Statistical analysis

Data were analyzed by means of StaView for Windows (SAS Inc v. 5.0.1, Cary, NC, USA). To measure the association level, Odds Ratio (OR) and the 95% corresponding test-based Confidence Interval (CI) were calculated. T-Student test was used to calculate significant differences between cases and controls at baseline for ordinal variables. Chi-square test was used to assess statistical differences among categorical variables. In all of evaluations *p*-values = 0.05 were considered statistically significant.

## Results

**Figure 1:**
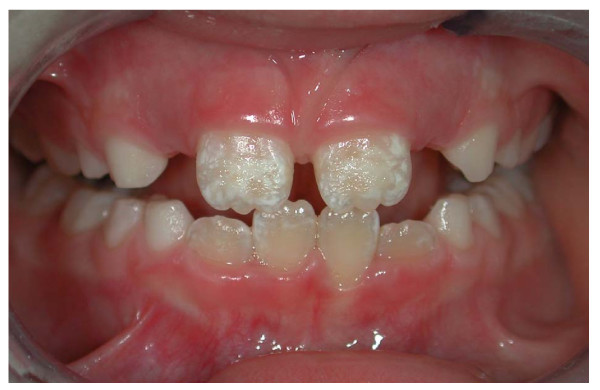
Symmetrical enamel hypoplasia of grade I on permanent incisors in a CD patient.

Enamel alterations were observed in 13/50 (26%) subjects with CD and in 8/50 (16%) controls, with a ratio male-female of 1:2 for the celiac group and 2:1 for control group (p > 0.2; OR = 1.8446; 95% CI = 0.6886: 4.9414). With respect to the severity score of hypoplasia, 10/13 CD patients showed lesions of degree 1 and 3/13 degree 2, in controls all were in degree 1. The grade 1 enamel defects were generally localized on incisor surfaces (for the anterior sectors) (Figure 1) and cuspid surfaces (for the posterior sectors), with dimensions from 1 to 3 mm and with a round-oval form, while that of grade 2 were on the canine and premolar vestibular surface. The colour alterations were white-yellowish, with clear margins, opaque and smooth surface.

**Figure 2:**
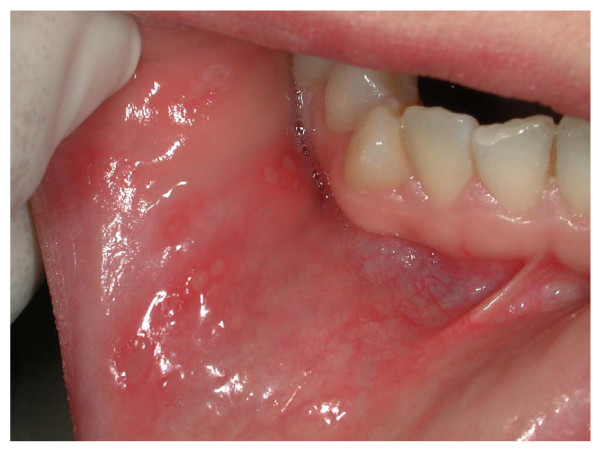
Several RAS on buccal mucosa in a CD patient.

Episodes of RAS occurred in 36% of CD patients (18/50) vs 12% of controls (6/50) (p = 0.0091; OR = 4.125; 95% CI = 1.4725: 11.552) with a male-female ratio of 1:1 and 2:3, respectively (Figure 2). In CD patients RAS showed greater rate of recurrence than in controls. Atrophic glossitis was reported in 4 cases and one control, and dermatitis herpetiformis in one patient with CD and none of subjects without CD.

## Discussion and conclusion

Recent epidemiology data showed the prevalence of CD to approach 1% of the general population [31-34]. However, the clinical presentation of CD seems to differ from the typical form observed in past years, as almost 50% of the patients with newly diagnosed CD do not present with gastrointestinal symptoms [35,36]. Thus, in order to identify the greatest number of "atypical" or "silent" CD patients and prevent long-term complications, it has been suggested that the clinicians should investigate those subjects who present "indirect" signs of CD, such as chronic anaemia [37], hyper-transaminasemia or hyperamylasemia of unknown origin [38,39], osteoporosis [40], autoimmune thyroid disorders [41].

As abnormalities of the oral cavity have been reported in CD, non-invasive clinical examination of the oral cavity can contribute to identify patients with atypical or silent CD [13,14,17,18,42].

As regards to changes of dental tissues, we did not found CD patients more likely to suffer from systematic and symmetric enamel defects. Indeed, a wide range of frequencies of enamel defects in CD patients has been reported in other studies [17,43-48]; our data are in agreement with other studies performed in Italy (Table 1) and the high frequency of enamel defect found in controls, as well as its severity, is likely to be related to environmental, dietetic and genetic factors [46]. Further studies are warranted to clarify the pathogenesis of this defect as nutritional, immunologic or genetic factors (association with the HLA DR3 allele) has been hypothesized [45,49]. With regard to celiac patients, enamel defects have been correlated to an altered phosphate-calcium metabolism and/or formation of antibodies against the matrix of enamel organ. The antigen correlated to class II molecules of the MHC could prime an immunity movement against the enamel organ, from which a mineralization disorder could derive [18]. In addiction, there is no strong evidence that these anomalies are correlated with the nutritional status, vitamin D deficiency or to an excess of fluoride incorporation. Current evidence suggests that an autoimmune pathogenesis is more likely, as enamel defects are also present in autoimmune diseases, such as some polyendocrine syndromes [46].

**Table 1:** Prevalence (%) of the dental enamel defects in CD patients

**Authors**	***n *CD patients**	**Prevalence %**
Aine 1996. [57]	86	96
Aine et al, 1990. [17]	40	83
Petrecca et al, 1994.* [18]	29	76
Aine et al, 1992. [58]	30	58.3
Aguirre et al,1997. [59]	137	52.5
Rasmusson et al, 2001. [8]	40	50
Balli et al, 1988.* [14]	111	34.7
Prati et al, 1987.* [60]	10	33.3
Martelossi et al., 1996. [61].*	603	32.4
Mariani et al, 1994.* [45]	84	28
***Present study, 2007***	***50***	***26***
Bucci et al, 2006.* [19]	72	20
Andersson-Wenckert et al, 1984. [13]	24	21
Lahteenoja et al, 1998. [22]	128	10.1

* = study performed among Italian individuals

With respect to oral soft lesions, we confirmed that CD patients are likely to suffer from RAS compared with healthy controls, especially before the gluten-free diet.

In our celiac population RAS was found in 26 % of CD patients with an OR of 4.12 in comparison with the controls. Even if a wide range of frequencies have been reported (Table 2) our data show the highest prevalence of RAS with respect to other Italian studies.

**Table 2:** Prevalence (%) of RAS in CD patients

**Authors, years**	**Number of CD patients**	**Prevalence (%)**
Sedghizadeh et al, 2002 [15]	61	41.0
***Present study, 2007***	***50***	***36.0***
Bucci et al., 2006 [19]*	72	33.3
Andersson-Wenckert 1984 [13]	19	26.3
Sood et al, 2003 [21]	96	19.8
Petrecca et al, 1994 [18] *	29	17.0
Majorana et al, 1992 [20]*	113	16.8
Lähteenoja et al, 1998 [22]	128	3.7

* = study performed among Italian individuals

In agreement with Sedghizadeh et al. [14], we suggested to consider RAS as a "risk indicator" of CD more than CD as a risk factor for RAS, although no definitive statement is possible on their predictive role for CD.

In addition the term "recurrent aphthous stomatitis" should be reserved to recurrent oral ulcer that present in patients without systemic diseases, while ulcers that have a clinical appearance similar to RAS, but found in patients with systemic disorders (such as CD) should be termed "aphthous-like ulcers" [50]. Even if the diagnostic criteria of RAS used in this study (namely, medical history and/or presence of detectable lesions) may represent a major limitation of present research, it is well accepted that recurrent and episodic nature of oral ulcerations requires medical history to be an important part of the diagnostic process.

RAS is often associated to haematinic (iron, folate, vitamin B12) deficiency [51,52]; since atypical or latent CD may not manifest itself with gastrointestinal signs/symptoms but often with iron/folate deficiency [53-56] we suggest that when patients show persistent RAS they should be examined for haematinic deficiencies. Only if one or more of these deficiencies are present, they should be screened for CD.

In conclusion, our data from central Italy confirming the higher prevalence of RAS or aphthous-like ulcers in patients with CD validate the hypothesis of their pathogenetic predisposition to oral mucosal lesions more than hard dental tissue lesions; further investigations are warranted to clarify the predictive role of these lesions in screening oligosymptomatic or asymptomatic CD.

## References

[B1] Korponay-Szabo IR, Kovacs JB, Czinner A, Goracz G, Vamos A, Szabo T (1999). High prevalence of silent celiac disease in preschool children screened with IgA/IgG antiendomysium antibodies. J Pediatr Gastroenterol Nutr.

[B2] Hill ID, Bhatnagar S, Cameron DJ, De Rosa S, Maki M, Russell GJ, Troncone R (2002). Celiac disease: Working Group Report of the First World Congress of Pediatric Gastroenterology, Hepatology, and Nutrition. J Pediatr Gastroenterol Nutr.

[B3] Catassi C, Ratsch IM, Fabiani E, Ricci S, Bordicchia F, Pierdomenico R, Giorgi PL (1995). High prevalence of undiagnosed coeliac disease in 5280 Italian students screened by antigliadin antibodies. Acta Paediatr.

[B4] Kolho KL, Farkkila MA, Savilahti E (1998). Undiagnosed coeliac disease is common in Finnish adults. Scand J Gastroenterol.

[B5] Carlsson AK, Axelsson IE, Borulf SK, Bredberg AC, Ivarsson SA (2001). Serological screening for celiac disease in healthy 2.5-year-old children in Sweden. Pediatrics.

[B6] Not T, Horvath K, Hill ID, Partanen J, Hammed A, Magazzu G, Fasano A (1998). Celiac disease risk in the USA: high prevalence of antiendomysium antibodies in healthy blood donors. Scand J Gastroenterol.

[B7] Pastore L, De Benedittis M, Petruzzi M, Tato D, Napoli C, Montagna MT, Catassi C, Serpico R (2004). [Importance of oral signs in the diagnosis of atypical forms of celiac disease]. Recenti Prog Med.

[B8] Rasmusson CG, Eriksson MA (2001). Celiac disease and mineralisation disturbances of permanent teeth. Int J Paediatr Dent.

[B9] Somech R, Spirer Z (2002). Celiac disease: extraintestinal manifestations, associated diseases, and complications. Adv Pediatr.

[B10] Green PH, Fleischauer AT, Bhagat G, Goyal R, Jabri B, Neugut AI (2003). Risk of malignancy in patients with celiac disease. Am J Med.

[B11] Tomasi TB Jr LL (1980). Mucosal immunity: The origin and migration patterns of cells in the secretory system.. J Allergy Clin Immunol.

[B12] Wray D (1981). Gluten-sensitive recurrent aphthous stomatitis. Dig Dis Sci.

[B13] Andersson-Wenckert I, Blomquist HK, Fredrikzon B (1984). Oral health in coeliac disease and cow's milk protein intolerance. Swed Dent J.

[B14] Balli MP, Balli ME, Mengoli M, Balli C, Balli F (1988). [Growth, skeletal and dental age in chronic diarrhea in childhood]. Pediatr Med Chir.

[B15] Sedghizadeh PP, Shuler CF, Allen CM, Beck FM, Kalmar JR (2002). Celiac disease and recurrent aphthous stomatitis: a report and review of the literature. Oral Surg Oral Med Oral Pathol Oral Radiol Endod.

[B16] Aine L (1986). Dental enamel defects and dental maturity in children and adolescents with coeliac disease. Proc Finn Dent Soc.

[B17] Aine L, Maki M, Collin P, Keyrilainen O (1990). Dental enamel defects in celiac disease. J Oral Pathol Med.

[B18] Petrecca S, Giammaria G, Giammaria AF (1994). [Oral cavity changes in the child with celiac disease]. Minerva Stomatol.

[B19] Bucci P, Carile F, Sangianantoni A, D'Angio F, Santarelli A, Lo Muzio L (2006). Oral aphthous ulcers and dental enamel defects in children with coeliac disease. Acta Paediatr.

[B20] Majorana A, Sapelli PL, Malagoli A, Meini A, Pillan MN, Duse M, Ugazio AG (1992). [Celiac disease and recurrent aphthous stomatitis. The clinical and immunogenetic aspects]. Minerva Stomatol.

[B21] Sood A, Midha V, Sood N, Malhotra V (2003). Adult celiac disease in northern India. Indian J Gastroenterol.

[B22] Lahteenoja H, Toivanen A, Viander M, Maki M, Irjala K, Raiha I, Syrjanen S (1998). Oral mucosal changes in coeliac patients on a gluten-free diet. Eur J Oral Sci.

[B23] Economopoulou P, Laskaris G (1986). Dermatitis herpetiformis: oral lesions as an early manifestation. Oral Surg Oral Med Oral Pathol.

[B24] Ventura A, Magazzu G, Greco L (1999). Duration of exposure to gluten and risk for autoimmune disorders in patients with celiac disease. SIGEP Study Group for Autoimmune Disorders in Celiac Disease. Gastroenterology.

[B25] Iltanen S, Collin P, Korpela M, Holm K, Partanen J, Polvi A, Maki M (1999). Celiac disease and markers of celiac disease latency in patients with primary Sjogren's syndrome. Am J Gastroenterol.

[B26] Scully C, Porter SR, Eveson JW (1993). Oral lichen planus and coeliac disease. Lancet.

[B27] Bhatnagar S, Tandon N (2006). Diagnosis of celiac disease. Indian J Pediatr.

[B28] van Heel DA, West J (2006). Recent advances in coeliac disease. Gut.

[B29] Stanley HR (1972). Aphthous lesions. Oral Surg Oral Med Oral Pathol.

[B30] Fasano A, Berti I, Gerarduzzi T, Not T, Colletti RB, Drago S, Elitsur Y, Green PH, Guandalini S, Hill ID, Pietzak M, Ventura A, Thorpe M, Kryszak D, Fornaroli F, Wasserman SS, Murray JA, Horvath K (2003). Prevalence of celiac disease in at-risk and not-at-risk groups in the United States: a large multicenter study. Arch Intern Med.

[B31] Tommasini A, Not T, Kiren V, Baldas V, Santon D, Trevisiol C, Berti I, Neri E, Gerarduzzi T, Bruno I, Lenhardt A, Zamuner E, Spano A, Crovella S, Martellossi S, Torre G, Sblattero D, Marzari R, Bradbury A, Tamburlini G, Ventura A (2004). Mass screening for coeliac disease using antihuman transglutaminase antibody assay. Arch Dis Child.

[B32] Maki M, Mustalahti K, Kokkonen J, Kulmala P, Haapalahti M, Karttunen T, Ilonen J, Laurila K, Dahlbom I, Hansson T, Hopfl P, Knip M (2003). Prevalence of Celiac disease among children in Finland. N Engl J Med.

[B33] Accomando S, Cataldo F (2004). The global village of celiac disease. Dig Liver Dis.

[B34] Maki M, Kallonen K, Lahdeaho ML, Visakorpi JK (1988). Changing pattern of childhood coeliac disease in Finland. Acta Paediatr Scand.

[B35] Pare P, Douville P, Caron D, Lagace R (1988). Adult celiac sprue: changes in the pattern of clinical recognition. J Clin Gastroenterol.

[B36] Carroccio A, Iannitto E, Cavataio F, Montalto G, Tumminello M, Campagna P, Lipari MG, Notarbartolo A, Iacono G (1998). Sideropenic anemia and celiac disease: one study, two points of view. Dig Dis Sci.

[B37] Bardella MT, Fraquelli M, Quatrini M, Molteni N, Bianchi P, Conte D (1995). Prevalence of hypertransaminasemia in adult celiac patients and effect of gluten-free diet. Hepatology.

[B38] Carroccio A, Di Prima L, Scalici C, Soresi M, Cefalu AB, Noto D, Averna MR, Montalto G, Iacono G (2006). Unexplained elevated serum pancreatic enzymes: a reason to suspect celiac disease. Clin Gastroenterol Hepatol.

[B39] Kemppainen T, Kroger H, Janatuinen E, Arnala I, Kosma VM, Pikkarainen P, Julkunen R, Jurvelin J, Alhava E, Uusitupa M (1999). Osteoporosis in adult patients with celiac disease. Bone.

[B40] Ventura A, Neri E, Ughi C, Leopaldi A, Citta A, Not T (2000). Gluten-dependent diabetes-related and thyroid-related autoantibodies in patients with celiac disease. J Pediatr.

[B41] Meini A, Pillan MN, Plebani A, Ugazio AG, Majorana A, Sapelli PL (1993). High prevalence of DRW10 and DQW1 antigens in celiac disease associated with recurrent aphthous stomatitis. Am J Gastroenterol.

[B42] Aine L (1986). [Dental enamel defects and dental maturity in children and adolescents with celiac disease]. Proc Finn Dent Soc.

[B43] Ventura A, Martelossi S (1997). Dental enamel defects and coeliac disease. Arch Dis Child.

[B44] Mariani P, Mazzilli MC, Margutti G, Lionetti P, Triglione P, Petronzelli F, Ferrante E, Bonamico M (1994). Coeliac disease, enamel defects and HLA typing. Acta Paediatr.

[B45] Rea F, Serpico R, Pluvio R, Busciolano M, Iovene A, Femiano F, Sessa G, Belnome G (1997). [Dental enamel hypoplasia in a group of celiac disease patients. Clinico-epidemiologic correlations]. Minerva Stomatol.

[B46] Ciccarelli NP DDF, Ped RI (1993). Lipoplasia dello smalto dentario dei denti permanenti di soggetti celiaci in challenge con glutine..

[B47] Mariani P FE, Ped. RI (1993). Difetti dello smalto dentario in un gruppo di bambini e adolescenti celiaci italiani..

[B48] Maki M, Aine L, Lipsanen V, Koskimies S (1991). Dental enamel defects in first-degree relatives of coeliac disease patients. Lancet.

[B49] Scully C (2006). Clinical practice. Aphthous ulceration. N Engl J Med.

[B50] Scully C, Felix DH (2005). Oral medicine--update for the dental practitioner. Aphthous and other common ulcers. Br Dent J.

[B51] Jurge S, Kuffer R, Scully C, Porter SR (2006). Mucosal disease series. Number VI. Recurrent aphthous stomatitis. Oral Dis.

[B52] Trier JS (1991). Celiac sprue. N Engl J Med.

[B53] Maki M, Collin P (1997). Coeliac disease. Lancet.

[B54] Catassi C, Fasano A (2002). New developments in childhood celiac disease. Curr Gastroenterol Rep.

[B55] Green PH, Barry M, Matsutani M (2003). Serologic tests for celiac disease. Gastroenterology.

[B56] Aine L (1996). Coeliac-type permanent-tooth enamel defects. Ann Med.

[B57] Aine L, Maki M, Reunala T (1992). Coeliac-type dental enamel defects in patients with dermatitis herpetiformis. Acta Derm Venereol.

[B58] Aguirre JM, Rodriguez R, Oribe D, Vitoria JC (1997). Dental enamel defects in celiac patients. Oral Surg Oral Med Oral Pathol Oral Radiol Endod.

[B59] Prati C, Santopadre A, Baroni C (1987). [Delayed eruption, enamel hypoplasia and caries in childhood celiac disease]. Minerva Stomatol.

[B60] Martelossi S, Torre G, Zanatta M, Del Santo M, Not T, Clarich G, Radovich F, Ventura A (1996). Dental enamel defects and screening for coeliac disease. Pediatr Med Chir.

